# Implementations and strategies of telehealth during COVID-19 outbreak: a systematic review

**DOI:** 10.1186/s12913-022-08235-4

**Published:** 2022-06-28

**Authors:** Stefania De Simone, Massimo Franco, Giuseppe Servillo, Maria Vargas

**Affiliations:** 1 Department of Political Sciences, University of Naples Federico II, Largo S. Marcellino, Naples, Italy; 2 Department of Neurosurgical, Reproductive and Odontostomatological Sciences, University of Naples “Federico II”, Via Pansini, Naples, Italy

**Keywords:** Telehealth, Strategies, COVID-19 outbreak, Review, Healthcare organization

## Abstract

**Background:**

Telehealth is an effective option to fight the outbreak of COVID-19. This review aims to systematically characterize the utilization and applications of telehealth during the COVID-19 pandemic focusing mainly on technology implementations.

**Methods:**

This study was conducted in accordance with Preferred Reporting Items for Systematic reviews and Meta-Analyses (PRISMA). The literature search was conducted in Science Direct, IEEE XPLORE, Scopus, and Web of Science databases from January 2020 until July 2021, with an English language restriction. A quality assessment was based on the Critical Appraisal Skills Programs checklist.

**Results:**

The included studies focused on the implementation of technology for telehealth, multidisciplinary approach, service satisfaction, guidelines, and medical training. They provided illustrative insight into the strategy of telehealth in different medical specialties, such as pediatric gastroenterology, oncology, ophthalmology, and laryngology. Nonsurgical specialties had the greatest number of telehealth visits. Clinicians showed positive attitudes toward the implementation of video telehealth visits; patients report high levels of satisfaction with this care and strong interest in continuing this modality as a significant portion of clinical practice.

**Conclusions:**

This systematic review provided an illustrative insight into the strategy of telehealth for different purposes. According to our findings, telehealth may be used in different medical area with a clear strategy of intervention according to patients’ and doctors’ needs.

**Supplementary Information:**

The online version contains supplementary material available at 10.1186/s12913-022-08235-4.

## Background

During this pandemic, healthcare organizations developed appropriate traits of flexibility and innovation to deal with institutional pressures [1–3]. The coronavirus disease 2019 (COVID-19) pandemic imposed the need for social distancing by also interrupting the hospital services. In response to this, innovations using information technologies were largely used within healthcare organizations [1].

Telehealth is a complex digital innovation that involves various stakeholders, across professional and organizational boundaries, with a multidisciplinary approach to ensure health care services to patients. Telehealth is the IT-enabled provision of medical services without in-person interactions between physicians and patients [4]. Through remote monitoring of patients, telehealth works as a preventative measure to avoid emergency department and hospital admissions and reduce costs by enabling a fast and accurate response to patients’ needs [5]. Indeed, while doctors take care of patients, the monitoring can be delegated to nurses or even to the patients themselves [5].

Telemedicine proved to be an effective strategy during the pandemic allowing the patient to connect in real time with health care providers despite the need for social distancing. Thus, this review aims to systematically characterize the utilization of telehealth and its applications during the COVID-19 pandemic focusing mainly on technology implementations.

## Methods

This study was conducted in accordance with Preferred Reporting Items for Systematic reviews and Meta-Analyses (PRISMA) [6]. A systematic search of the literature in Sciencedirect, IEEE XPLORE, Scopus and Web of Science databases was performed from January 2020 until July 2021. The following keywords were used: (‘Telehealth’ _OR ‘e-health’ _OR ‘Telecare’ _OR ‘Telehealth’ _OR ‘remote monitoring’ _OR ‘mHealth’ _OR ‘Medical system’ _OR ‘health care service’ _OR ‘Telemedicine’) AND (*Disease* OR *Infection* OR *Virus* OR *Epidemic* OR *Outbreak* OR *Pandemic* OR *COVID-19* OR *COVID-19* OR *SARS-COV-2*).

Limited data existed on the telehealth application in COVID-19 since the recent onset of the pandemic. To collect all existing evidences on this topic, we plan to include primary studies such as RCTs, prospective cohort studies, retrospective studies and all kind of reviews published in English language on technologies implementation for telehealth in COVID-19 and non-COVID-19 patients. Conference paper and articles not in English language were excluded.

### Data extraction, quality assessment and quantitative analysis

Data were independently extracted from each study by two authors (MV and SDS) using a data recording form developed for this purpose.

Two pairs of independent reviewers performed the initial selection to screen titles and abstracts (MV, SDS). For detailed evaluation, a full-text copy of relevant studies was obtained. Using a pre-standardized data extraction form, paired reviewers (MV, SDS) extracted the data from each study.

Title, year, type of study, setting, aim, strategy/type of telehealth, personnel involved, outcomes and main findings of included studies were considered data of interest for this systematic review.

Two reviewers (MF, GS) checked the accuracy of data extracted and further evaluated the quality of included studies. The Critical Appraisal Skills Programs checklist was used as quality assessment checklist; it included 11 criteria [7] to ensure the quality of the included studies. Each assessed criteria could be assigned a quality score of 0 for ‘does not meet’, 0.5 for ‘partially meet’ and 1 for ‘fully meet’. The total quality score of each article ranges from 0 to 11. According to this, a signified high-quality article is defined by a high score. Any possible disagreement on data extraction and quality assessment was solved through consultation with an external reviewer, if needed.

For the purpose of quantitative analysis, we planned to collect the number of visits and possible quantitative outcomes reported by the included studies.

## Results

**Fig.1:**
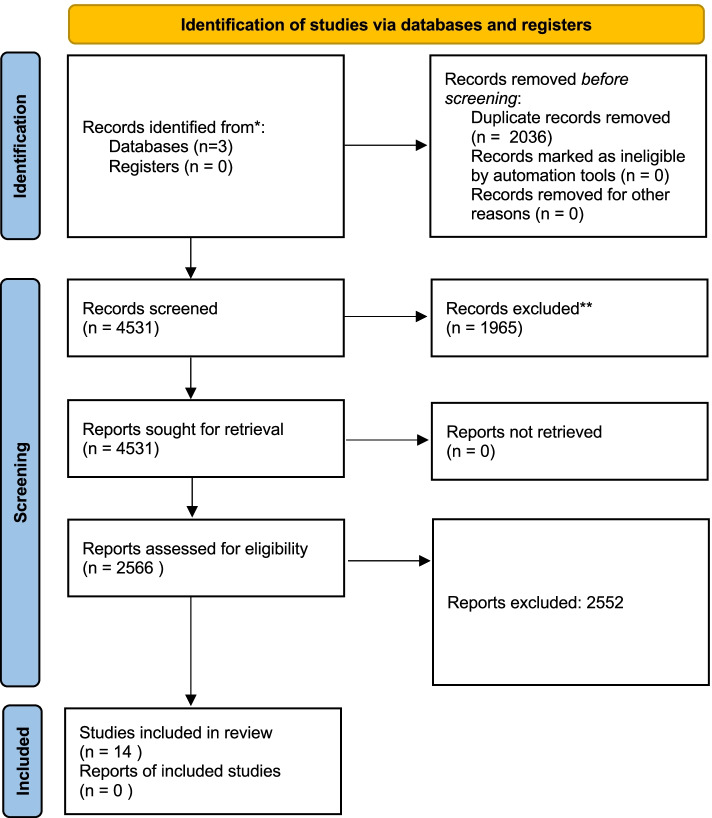
PRISMA flow chart of included studies

A total of 6567 records were identified across the different databases. After the screening process, 14 articles related to technology, telehealth, and COVID-19 were included (Fig. 1).

During the quality evaluation process, three studies reached a score of 9.5 points, three studies 9 points, three studies 8.5 points, five studies reached a score ≤ 7 points (Table 1).

**Table 1:** Quality of included studies checked by the Critical Appraisal Skills Programs checklist. Quality score of 0 for ‘does not meet’, 0.5 for ‘partially meet’ and 1 for ‘fully meet’ may be assigned to each assessed criteria. The total quality score of each article ranges from 0 to 11

Reference	Q1 Aim	Q2 Method	Q3 Research Methods	Q4 Settings & Sample	Q5 Measures Definition	Q6 Measures	Q7 Data Collection	Q8 Data Analysis	Q9 Comparison	Q10 Findings	Q11	Total
Berg	1	1	0	0	0	0	0	1	0	1	0	4
Saleem	1	1	1	0.5	0.5	0.5	1	1	0	1	0	7.5
Hron	1	1	1	1	1	1	1	1	0	1	0.5	9.5
Strohl	1	1	1	1	0.5	0.5	1	1	0	1	1	9
Cassar	1	1	1	0.5	0	1	0.5	1	0	1	0	7
Gentry	1	1	1	1	0.5	0.5	1	1	0.5	1	1	9.5
Smith	1	1	1	1	0	1	1	1	0	1	1	9
Goenka	1	1	1	1	0	1	1	1	0	1	0.5	8.5
Checcucci	1	1	1	1	0	0.5	1	1	0	1	1	8.5
Franciosi	1	1	1	1	0	1	1	1	0	1	1	9
Harris	1	1	1	1	0.5	1	1	1	0	1	0	8.5
Leite	1	1	1	0	0	0	0	1	0	1	1	6
Cerqueira	1	1	1	1	0.5	1	1	1	0	1	1	9.5
Basil	1	1	0.5	0.5	0	1	0.5	1	1	1	1	8.5

**Fig. 2:**
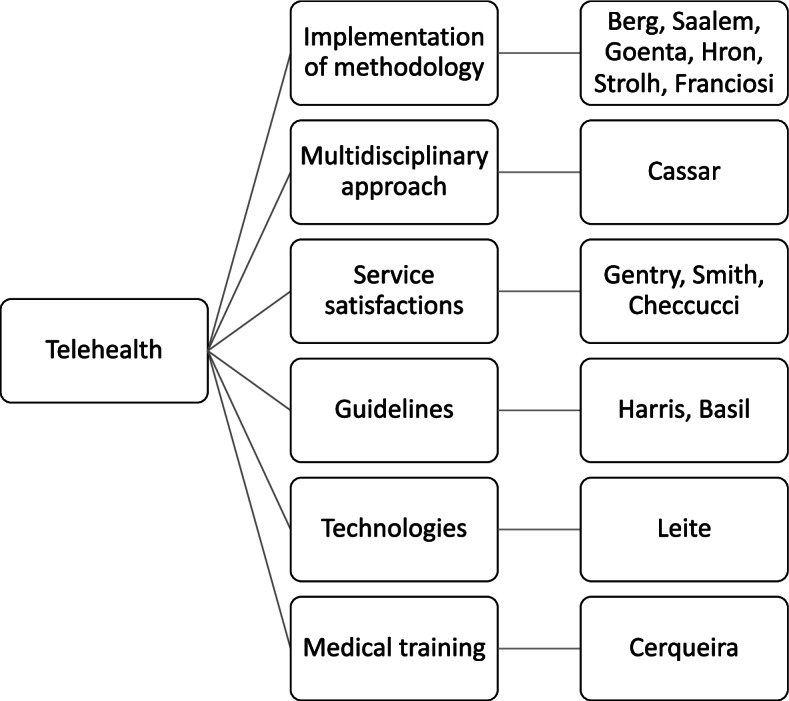
Categories of telehealth evaluated in the included studies

Figure 2 summarized the category of telehealth evaluated in the included studies.

Six studies focused on the implementation of technology for telehealth [8–13]. Berg et al. [8], Saleem et al. [9], Goenka et al. [10], Hron et al. [11], and Strol et al. [12] discussed the usefulness of telehealth during the COVID-19 in different medical specialties, such as pediatric gastroenterology, ophthalmology, radiation oncology, inpatient clinics and laryngology. Berg et al. [8] found that telehealth may improve clinical outcome in children with inflammatory bowel disease. Saleem et al. [9] reported the implementation of a workflow diagram that maps the ophthalmology telehealth visit process with the aim to adapt it for the daily evaluation. Goenka et al. [10] found that the 2-way audio telehealth visits were associated with lower billing codes compared with in-person visits. Horn et al. [11] reported that the host of 1820 inpatients, for a total amount of 104 647 min of telehealth, were sufficient to build rapport and to perform a reasonable clinical examination. Strol et al. [12] discussed the key areas to implement the telehealth visits in a tertiary-care laryngology practice. They stated that the key areas were the set-up of the visit, patient examination and treatment, optimization of the tele-visit, limitations of the tele-visit and reimbursement considerations [12]. Franciosi et al. [13] reported that telehealth is an essential tool with the potential to improve access to health care, particularly in nonprocedural specialties. The authors [13] showed the potential shortcomings of telemedicine services for non-English speaking patients and the increased number of telehealth visits for nonsurgical specialties. Cassar et al. [14] reported the experience of using a team called the ‘community covid-19 initial assessment team’ in managing covid-19 patients. They found that the use of telehealth visits did not increase the morbidity and mortality of infected patients [14]. Three studies focused on the service satisfaction [15–17]. Gentry et al. [15] showed the high satisfaction, acceptability, feasibility and appropriateness of mental health clinicians while using video telehealth visits. Smith et al. [16] highlighted the positive attitude of women underwent fetal ultrasound telemedicine service and the consequent reduction in family costs and journey times. Checcucci et al. [17] reported the high appreciation of patients suffering from benign urological diseases, referring to phone-call visit (phone counselling) as useful telemedicine tool.

Two studies provided guidelines to healthcare workers [18, 19]. Harris et al. [18] reported systematic protocols for telehealth intervention in post-acute and long-term care facility residents in order to reduce mortality and hospitalization rates. Basil et al. [19] highlighted the effectiveness of telehealth visits by reporting the incidence of the conversion to in-person visit for only 26 out of 2157 telehealth visits. The authors [19] provided guidelines to perform and standardize the telehealth for neurological examination. One study focused on technology [20] by discussing the strategic role of telehealth in managing the COVID-19 pandemic to relieve congested health-care facilities and avoid the risk of further infection. The author reported the effectiveness of a 3-T model, that is tracking, testing and treating, to defeat the spread of COVID-19. One study highlighted the medical training [21]. In particular Cerqueira-Silva et al. [21] described a strategy combing telehealth and medical training to mitigate the adverse effects of the COVID-19 pandemic. Patients staying at home received a guidance to avoid disease transmission and reduce the spread of pandemic.

Table 2 summarized study design, setting, aim, type of telehealth strategy used, personnel involved and outcome/main finding of the included studies.

**Table 2:** Summary characteristic of included study in literature review

Studies, Years	Study design	Setting	Aim	Strategy/type of telehealth	Personnel involved	Outcomes/ findings
Berg et al., 2020 [8]	Not reported	Pediatric gastroenterology	Discussing implementation of telehealth during COVID-19 pandemic	Telehealth visits Virtual check-in via telephone and audiovisual application, E-visits through an online patient portal	Physicians and nonphysician healthcare providers	Practice recommendations for introducing and expanding telehealth
Saleem et al., 2020 [9]	Review	Ophthalmology	Discussing telehealth implementation methodologies during COVID-19 pandemic	Telehealth visit (telephone calls) Virtual check-in Digital encounters	Physicians	Teleophthalmology model mapping the telehealth visit cycle
Goenka et al., 2021 [10]	Retrospective review of cases	Radiation oncology	Implementing a telehealth service during the COVID-19 pandemic	Audio–video and telehealth platform	Physicians	Decrease in billable activity Reduction of in-person visits (from 100 to 21%)
Hron et al., 2020 [11]	Not reported	Inpatient clinics	Evaluating the implementation of telehealth program in response to COVID-19 pandemic	Videoconferencing system	Physicians and nursing clinical informatics experts	Usefulness of telehealth to perform physical exam, resulting from 1.820 inpatient telehealth sessions (13.3 sessions per 100 bedded days)
Strohl et al., 2020 [12]	Review	Laryngology	Implementing telemedicine during the COVID-19 Pandemic Learning experiences and implementation of telehealth during the COVID-19 pandemic	Video visit	Provider (ie, laryngologist and/or speech-language pathologist)	Key areas included (1) how to set up and structure a telemedicine visit and maintain patient confidentiality, (2) patient examination and treatment initiation, (3) optimization of the tele-visit, (4) recognition of when a tele-visit is insufficient for patient care needs, (5) billing/reimbursement considerations
Franciosi et al., 2021 [13]	Cross-sectional	Primary care, pediatric and adult surgical and non-surgical cares	Evaluating the impact of telehealth implementation on underserved populations	Televisits and digital platform	Providers	Changes in patient demographics in telehealth visit, including a younger population, fewer non-English-speaking patients The greatest number of telehealth visits in nonsurgical specialties
Cassar et al., 2021 [14]	Population-based study	COVID-19 evaluation	Implementing a telemedicine system to protect patients from COVID-19 transmission and to manage the infected patients	Teleworking system based on telephone communications, emails and a shared online database on portal platform	Experienced doctors, including emergency medicine, general medicine and geriatric medicine and an infectious disease specialist	Safely management of infected patients in the community No increased morbidity or mortality related to the medical decisions using this telemedicine tool
Gentry et al., 2021 [15]	Cross-sectional	Mental health	Examining clinician satisfaction with telehealth services during Covid-19 pandemic	Video telehealth visits	Psychiatrists, psychologists, and mental health counselors	High levels of acceptability, feasibility, and appropriateness of video telehealth in the opinions of clinicians High levels of satisfaction of clinicians with video telehealth visits
Smith et al., 2021 [16]	Collection of participant questionnaire data	Obstetrics, fetal ultrasound medicine	Evaluating women’s views of fetal ultrasound telemedicine and family costs	Video-conferencing	Fetal medicine specialist Fetal cardiology clinics	High levels of satisfaction of women with video telehealth visits Reduction in family costs and journey times
Checcucci et al., 2021 [17]	Collection of patient questionnaire data during call	Urology	Assessing the use of telemedicine to follow-up patients with benign urologic diseases during the COVID-19 pandemic	Phone-call visits (phone counselling)	Medical staff	High appreciation by patients for telemedicine (phone visit comprehensibility, usefulness and ease of communication of exams)
Harris et al., 2020 [18]	Not reported	Post-acute and Long-term care facility for patients with COVID-19	Reporting systematic protocols for guiding telehealth intervention during the COVID-19 pandemic	Daily multidisciplinary virtual rounds and telemedicine consultation Remote physical examination and videoconferencing	University physicians Telehealth engineers	Lower mortality and hospitalization rates: during a month, 18 out of 48 (38%) facility residents required hospitalization and 6 (12.5%) died No staff required hospitalization
Basil et al., 2021 [19]	Retrospective review	Neurosurgical evaluation	Providing guidelines to healthcare workers for performing neurological examination via telemedicine during the COVID-19 pandemic	Telehealth visits	Neurosurgeons	Effectiveness of telehealth visits: of 2157 telehealth visits performed in department’s outpatient clinic visits only 26 converted to in-person visits for a more detailed patient evaluation
Leite et al., 2020 [20]	Viewpoint	COVID-19 evaluation	Discussing the strategic role of telehealth technologies in managing the COVID-19 pandemic	Electronic and telecommunications technologies	Physicians	Telehealth technologies as a frontline ally to avoid the spread of the virus, by tracking, testing and treating
Cerqueira-Silva et al., 2021 [21]	Case study	COVID-19 evaluation	Describing strategy that combines telehealth and medical training to mitigate the adverse effects of COVID-19	Tele-screening	Medical students Physicians, including residents and medical doctors	Minimization of interpretation bias and rapid responses in unexpected situations Not required for users to possess education level or be digitally literate in order to access the service

For the quantitative purpose we were able to identify the amount of telehealth visit performed by each study (Table 1-supplemetary materials).

## Discussion

Three categories of telehealth can be identified by current literature: 1) telehealth visits, a medical visit using of audio and visual telecommunications, 2) virtual check-ins, a brief communication using telephone, audiovisual application, secure text messaging, e-mail, or a patient portal, 3) E-visits through an online patient portal [8].

Telehealth allows health care professionals to ask special questions, collect required information, triage of patient, and supply consultation while the patient is at home.

An interesting element emerging from this review is the large, estimated amount of telehealth visits in different specialties. Ten articles reported the number of telehealth visits performed during the study periods for a total of 176.414 medical consultations.

The studies included in this systematic review demonstrated the expansion of telemedicine across all medical specialties in many countries in response to a unique and sudden need for virtual medical visits created by the COVID-19 pandemic. Our findings, in line with the literature, showed that nonsurgical specialties have the greatest number of telehealth visits [11].

Telehealth may add potential benefit in non-emergency/routine areas and in services not requiring in person patient-doctor interaction. In addition, during COVID-19 pandemic, telehealth may have the potential role of delivering health care services for underserved populations by eliminating barriers such as transportation needs, distance from specialty providers, and time off from work [13].

Telemedicine may also improve health care delivery by substituting in-person care [4]. Remote care reduces the use of different resources in health centers and improves access to care while minimizing the risk of direct transmission of the infectious agent from person to person [22]. Most of the included studies showed the efficacy of telehealth system in drastically reducing the amount of time spent in the room with the patient per day since some portions of the physical exam were remotely performed. Patients and families appreciated minimizing contact with health care providers during a frightening time, and clinicians showed positive attitudes toward the implementation of telehealth visits, and also a strong interest in continuing this modality as a significant portion of clinical practice [15–17].

Telehealth is a promising tool that may modernize the traditional in-person clinical practice and inspire alternative ways of organizing or governing the economic activity of health care [23]. According to our findings, telehealth visits are suitable for follow-up visits after patients have already seen the doctor, exam of easy-to-see areas, like eyes or skin, counseling and other mental health services, prescription refills, and monitoring chronic conditions like diabetes or asthma. On the other hand, the in-person visits are better for the first visit, for clinical evaluation that needs a hands-on approach, blood tests, X-rays, and other imaging tests.

While clinical history may be taken in-person and by telehealth, physical examination, instrumental evaluation, and laboratory findings are far from being included in a visit from remote. With those premises we tried to identify a model guiding the use of telemedicine to set which phases of the diagnostic process should be done in person and which ones could rely on telehealth (Table 3).

**Table 3:** Phases of the Diagnostic process performed in-person or in telehealth

Diagnostic process	In-person	Telehealth
***1) Medical history***		
Information	X	X
Symptoms	X	X
Signs	X	X
**2) Physical examination**		
Inspection	X	X
Palpation	X	
Percussion	X	
Auscultation	X	
**3) Instrumental examinations**		
Invasive	X	
Non-invasive	X	X
**4) Laboratory investigations**		
Basic	X	X
Advanced	X	

During the COVID-19 pandemic, telehealth had the aim to screen for infected people, oversee affected subjects, and ensure continuity of care of chronically ill patients. However, as reported by this review, the use of telemedicine was not a homogeneous process [24]. This was due to differences in the awareness of the importance of telemedicine, variability in the quality of the infrastructures, level of informatics literacy of healthcare professionals and patients, and reimbursement schemes and plans. However, the experience collected during the COVID-19 pandemic may help to develop a more coordinated general strategy to favor the implementation of telehealth at large scale in the healthcare systems. In our opinion achieving this goal will be useful to help the healthcare system to be prepared for future pandemic and to develop virtual hospitals, home-base but telehealth-assisted, that may reduce the burden of conventional hospital.

## Conclusions

This systematic review provides an illustrative insight into the implementation of telehealth for different purposes. Telehealth may be used in different medical areas with a clear strategy of intervention according to the patients’ and doctors’ needs. As future perspective, we suggest the implementation of telehealth systems to build virtual hospitals, home-based but telehealth-assisted, to reduce the burden of conventional hospital.

## Supplementary Information

**Additional file 1.**

## Data Availability

The datasets generated and analysed during the current study are not publicly available due [project dataset] but are available from the corresponding author on reasonable request.

## Abbreviation

COVID-19: Coronavirus disease 2019
