# Supplementary material for: Implementations and strategies of telehealth during COVID-19 outbreak: a systematic review
Source: BMC Health Serv Res. 2022 Jun 28;22:833. doi: 10.1186/s12913-022-08235-4 (PMC9238134; doi:10.1186/s12913-022-08235-4)
Supplement: Supplementary file 1 — Additional file 1. [file 12913_2022_8235_MOESM1_ESM.docx]

**Table 1 – supplementary materials. Main specialties and amount of telehealth visit performed by the included studies.**

| **Study** | **Main Specialties/ward** | **Amount of telehealth visits** |
| --- | --- | --- |
| Berg | Pediatric gastroenterology | NA |
| Basil | Neurosurgical evaluation | 2157 |
| Cassar | COVID-19 evaluation | 369 |
| Cerqueira | COVID-19 evaluation | 111 965 |
| Checcucci | Telehealth for benign urologic condition | 607 |
| Franciosi | Primary care, pediatric and adult/surgical and non-surgical cares | 45933 |
| Gentry | Mental health evaluation | 10256 |
| Goenka | Radiation oncology | 2997 |
| Harris | Long term care facility patients with COVID-19 | 13 |
| Hron | Inpatient clinics | 1820 |
| Leite | COVID-19 evaluation | NA |
| Saleem | Ophthalmology | NA |
| Smith | Fetal ultrasound telemedicine | 297 |
| Strohl | Laryngology | NA |

**NA: not available**
